# Are play and screen time associated with British preschoolers’ mental health? Cross-sectional findings from the British Preschool Children’s Play Survey

**DOI:** 10.1136/bmjopen-2025-105101

**Published:** 2026-01-29

**Authors:** Kathryn R Hesketh, Helen F Dodd

**Affiliations:** 1 Behavioural Science and Health, UCL, London, UK; 2 MRC Epidemiology Unit, University of Cambridge, Cambridge, UK; 3 University of Exeter, Exeter, UK

**Keywords:** Cross-Sectional Studies, Community child health, Behavior, MENTAL HEALTH

## Abstract

**Abstract:**

**Objectives:**

To investigate associations between adventurous play, outdoor play and screen time and mental health (MH) in British preschool-aged children.

**Design:**

Cross-sectional.

**Setting:**

A nationally representative sample of caregivers of 2–4 years old (n=1066) in England, Scotland and Wales (Britain), recruited through an online research data and analytics group (YouGov UK).

**Participants:**

Caregivers of 1018 children provided valid complete-case data (age 2: n=298 (29%), age 3: n=365 (36%), age 4: n=355 (35%); female n=481 (47%); white: n=878 (81%)).

**Outcome measures:**

Four outcomes, derived from parent-report questionnaires: internalising and externalising scores (using the Strengths and Difficulties Questionnaire) and positive and negative affect scores (using the Positive and Negative Affect Schedule for Children-P). Linear regression was used to explore associations between the three exposures (time (in hours per week) a child spent: (1) playing adventurously; and engaging in (2) educational screen time and (3) recreational screen time) and the four outcomes; interactions between play and screen time variables were also tested. Models were adjusted for child and parental demographic variables.

**Results:**

For each additional hour per week a child engaged in adventurous play, they had lower internalising scores (−0.02 (−0.03 to –0.01)) and higher positive affect scores (0.04 (0.02 to 0.05)). More hours per day (vs <1 hour/day) of educational screen time and recreational screen time were associated with higher internalising and negative affect scores. Greater educational screen time was associated with lower positive affect and higher externalising scores, with adventurous play moderating the association between higher educational screen time, internalising and negative affect.

**Conclusion:**

In British preschoolers, adventurous play is associated with better MH outcomes, whereas higher educational screen time was associated with poorer MH, indicating that adventurous play may benefit preschoolers’ MH or that preschoolers with better mental health are more likely to engage in adventurous play. Adventurous play may also offset possible negative associations with screen time.

**STRENGTHS AND LIMITATIONS OF THIS STUDY**

- In this nationally representative population-based study, we assessed several types of play in 2–4 years old, including adventurous and outdoor play, outside of childcare.
- Participants were sampled from diverse geographical locations, socioeconomic strata and ethnic groups, with survey weights used to reduce the risk of sampling bias.
- It uses robust validated tools to assess children’s play and mental health outcomes, adjusting for parent mental health outcomes as evidence suggests that parental mental health may impact estimates of child mental health.
- While parent-report is frequently used as a means of collecting large-scale national data, it provides only one perception of a child’s behaviours and mental health outcomes.
- This cross-sectional data provides a helpful indication of associations between play, screen time and mental health, but longitudinal evidence is now required to elucidate causal mechanisms to inform future promotion efforts.

## Introduction

 Good mental health in early childhood lays the foundation for children’s learning, development and future health.^1^ In young children, ‘mental health’ refers to positive social development and emotional well-being from birth to age 5.^2^ Young children with poorer mental health may miss out on developmental experiences that promote early learning^3^ and have impaired interactions with peers or caregivers which, in turn, may impact their development.^4^ Good mental health in the preschool years is also associated with decreased risk for a mental health disorder in middle childhood.^5^ Ensuring that children have a positive start to life, and that their mental well-being and development are supported, can therefore have long-lasting benefits across the life course. This is vital considering recent statistics showing that more than 10% of UK children and young people (aged 5–16 years) now have a diagnosable mental health problem.^6^

Many social determinants influence children’s mental health, including socioeconomic circumstances, peer and parent relationships and children’s wider environment.^7^ Play is one such determinant: vital during the early years, it can help to strengthen bonds between children and caregivers, reduce stress, allow children to process difficult emotions and build confidence.^8^ Other factors, such as physical activity, which is mostly accrued through play in the early years, are also known to impact young children’s mental well-being.^9^ Active and outdoor play provides ‘health giving’ physical health benefits, while promoting positive mental health in children.^10^ Importantly, being outside can facilitate risky or adventurous play, defined as thrilling play with an element of danger.^11^ Recent evidence suggests that British preschool-aged children play adventurously for approximately ~22 hours per week, but girls and children from minority ethnic groups may have fewer opportunities for adventurous play, particularly in nature, compared with their peers.^12^ Moreover, adventurous play and mental health appear to be associated in school-aged children.^13^ If this same association exists in preschool-aged children, it is possible that the lower levels of adventurous play observed in some groups may compound inequalities in physical and mental health.

Despite the benefits of adventurous play, opportunities for this type of play are known to have decreased in older children, with a concurrent increase in the amount of time they now spend in front of a screen.^14^ The WHO recommends that preschool-aged children (1–4 years) have no more than 1 hour of screen time per day.^9^ With an explosion of devices available for young children to use, both positive and negative impacts on development have been reported. Devices and associated applications have significant potential to improve young children’s early reading, support creative thinking and promote social interaction with family members living elsewhere.^15^ Conversely, studies have demonstrated potential negative effects of increased screen time on children’s executive functioning, sensorimotor development and academic outcomes.^16^ This suggests that differing types of screen time (eg, educational, recreational) may have variable impacts on children’s health and well-being.^15^,^17^ In addition, emerging evidence suggests that volume of screen time may be important: higher levels of screen time (>1 hour/day) in USA 2–5 years old were shown to be associated with decreased psychological well-being, including less curiosity and self-control, lower emotional stability and being more difficult to care for.^18^ Screen time in younger children has also been associated with poorer physical health^19^ and poorer sleep when screens are used prior to bedtime.^20^ These associations are also apparent in older children and appear to persist through adolescence both cross-sectionally and prospectively,^21^ suggesting that it may be important to limit screen time in younger children for their wider and longer-term health and well-being.

Given the prospective association between good mental health during the preschool years and in later childhood, a better understanding of how early childhood behaviours impact mental health in under 5s is required. There is a relative absence of evidence about the link between screen time and mental health in UK preschool-aged children, and no study to date has assessed how types of play and screen time interact to influence mental health. Moreover, although both outdoor and adventurous play have been proposed to be important for children’s mental health, no research has directly explored the association between these subtly different types of play in preschool-aged children. We therefore aimed to explore the association between play, screen time and mental health outcomes in a nationally representative sample of 2–4 years old living in Britain. We investigated how total time spent playing outdoors or adventurously and engaging in screen time was associated with commonly used mental health indicators (ie, Strengths and Difficulties Questionnaire (SDQ) and Positive and Negative Affect Schedule (PANAS)) in these children. We hypothesised that greater adventurous play would be associated with better mental health, and more (recreational) screen time would be associated with poorer mental health. Finally, we conducted exploratory interaction analyses to determine whether play modified the association between screen time and mental health.

### Study design and setting

Data were from the British Preschool Children’s Play Survey (BPCPS), a nationally representative cross-sectional online survey, conducted to investigate play behaviours in UK preschool-aged children (2–4 years old). Based on the British Children’s Play Survey (BCPS) (focused on children aged 5–11 years^13^), the BPCPS survey was designed to closely align to the BCPS, to allow comparison across datasets. Detailed explanations of measures undertaken in the BPCPS are provided elsewhere.^12^ Sample size was pragmatic and driven by available funding; it is one of the largest studies to examine preschool-aged children’s play internationally.

### Procedure

**Figure 1:**
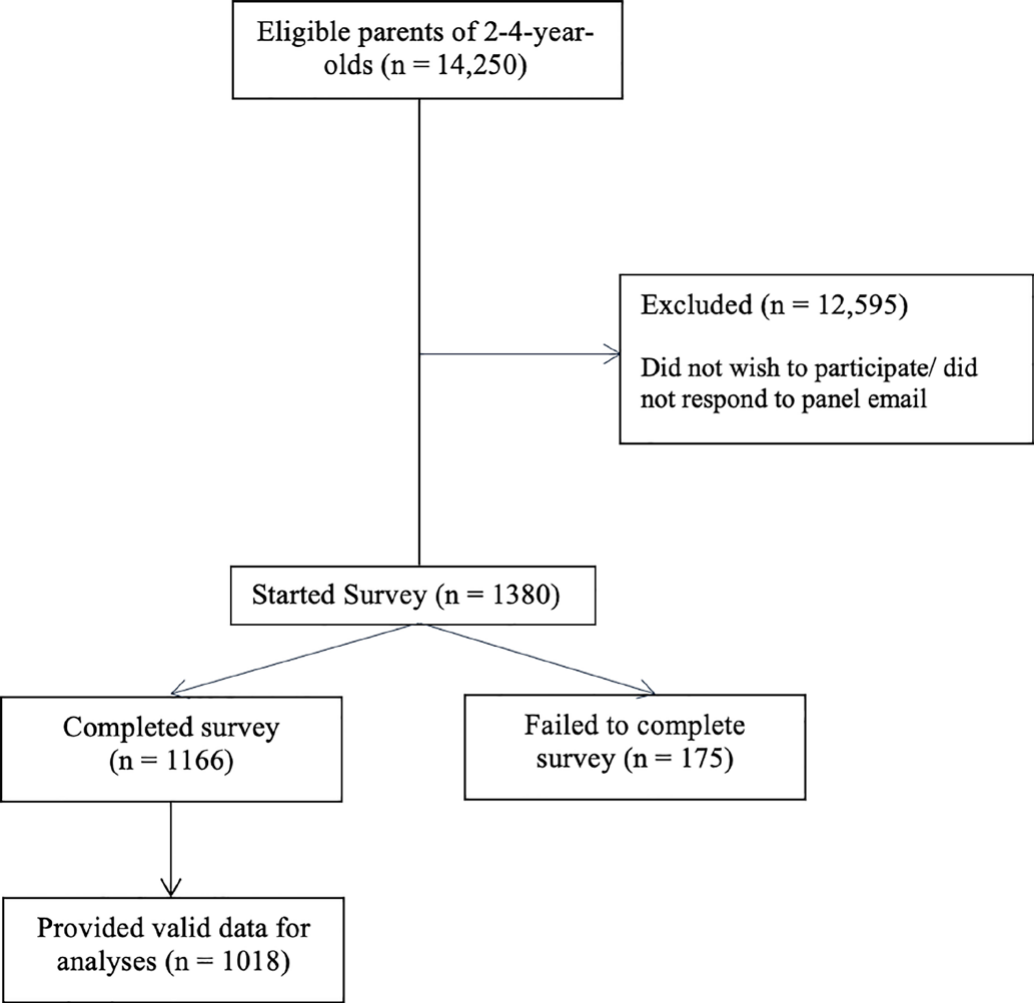
Flow of participants through each stage of study recruitment.

Data were collected by YouGov through their online panel, which includes over 1 million adults living in the UK. YouGov recruit panel members from a diverse range of sources to ensure diversity, and use active sampling, inviting members of their research panel via email to complete online surveys. According to YouGov records, 14 250 parents had a child aged 2–4 years and were eligible to participate (figure 1). On starting the online survey, participants were initially asked to indicate whether they were parents of children aged 2–4 years, and after obtaining electronic consent, respondents were provided with a link to access the survey. They were advised to respond about their eldest child in the age range. The survey included validated questionnaires about the mental health of children and parents, children’s play (time), including outdoor and adventurous play, childcare attendance, socio-demographic and geographical information. On completion of the survey, all respondents were given YouGov reward points which could be exchanged for vouchers or payments. For the present study, the participating sample was recruited to be approximately representative of the national population and then weighted based on age, gender, social class, region and level of education to the national profile of adults living in the UK (see below).

### Measures

#### Outcome measures

Child psychological well-being was assessed using two measures: the SDQ for parents of 2–4 years old (SDQ 2–4)^22 23^ and the Positive and Negative Affect Schedule for Children-P (PANAS-C/P).^24^

#### Strengths and Difficulties Questionnaire

The SDQ 2–4 is a 25-item screening questionnaire designed for parents and teachers of children aged 2–4 years. It asks about positive and negative attributes of the child, with items combined to create five subscales: emotional symptoms, conduct problems, hyperactivity/inattention, peer relationship problems and prosocial behaviour. We calculated an ‘internalising’ score by summing the emotional problems and peer relationship problems subscales, and an ‘externalising’ score by summing conduct problems and hyperactivity/inattention subscales. The prosocial behaviour subscale was not used for these analyses. The SDQ has been used widely in this age group and has strong psychometric properties (see www.sdqinfo.org^23^).

#### Positive and Negative Affect Schedule

PANAS-C/P is a 10-item scale, with care-providers asked to rate the extent to which their child has exhibited each of 10 emotions described in the past week; five items are summed to provide a positive affect score (PANASPA) and five remaining to give a negative affect score (PANASNA). The scale has good psychometric properties.^24^

### Exposure variables

#### Children’s play

The Children’s Play Scale (CPS)^25^ was developed to better understand play in school-aged children and was applied to the preschool-aged group here; after piloting with parents of preschool-aged children, no amendments were deemed necessary to capture play in younger age groups. Using the CPS, parents indicated how *frequently* (0–7 days) and for *how long* (in hours) their child played in a range of places, when not in childcare and not playing on a screen (in a home; outside at a home; at a playground; in green space (not a garden); in the street/public places; outdoors near water; and at indoor play centres and pools. Parents were asked to answer about frequency and length of time playing in each location for Autumn/Winter and Spring/Summer separately. These responses were then summed to derive the number of hours per year each child spent playing in each place. The CPS also has an adventurous play supplement, to capture more ‘risky’ play, which was used here. This asks parents to indicate how adventurously their child played at each play location using a scale from 1 (very low levels of adventure) to 5 (maximum levels of adventure). The CPS was created with input from experts and parents, including iterative revisions with the parent group. While the measure has not been formally validated against time use diaries or geographical information systems, it does have good face validity (as judged by parent piloting) and reliability.^25^

Responses were used to create two exposure variables: adventurous play and outdoor play. We chose to assess both exposures to differentiate between playing outside and playing adventurously, as most of the latter occurs during the former but they are not synonymous. To derive adventurous play, the time spent playing across all locations where the child was reported to play with at least a mild level of adventure (2 on the Likert scale) was summed. For outdoor play, the time children spent playing (at any adventure level) across all locations where the child was reported to play outside was summed. For both variables, we first derived a yearly total (in hours), which was then divided by 52 to give hours/week spent playing adventurously and outdoors.

#### Screen time

We applied a scale used previously with parents of preschool-aged children^26^ to assess screen time. This asked participants to report the amount their child spent looking at a screen (eg, television screen, tablet, phone) on weekdays and weekends (in hours per day). Considering the potential differential impacts on development, we asked participants to answer separately for (1) recreational/communication and (2) educational screen time. Care providers answered from: ‘No time (ie,0 minutes)’, ‘less than 1 hour’, ‘between 1 to 2 hours’, ‘between 2 to 3 hours’, ‘between 3 to 4 hours’, ‘more than 4 hours’ or ‘don’t know’. The midpoint value of each category (ie, 30 min for ‘less than 1 hour’) was used to derive an average daily total in hours per day, combining weekday and weekend values (in the ratio of 5:2). Children were then categorised into three groups: ‘<1 hour’, ‘1–2 hours’, ‘>2 hours’, for each of the two exposures, capturing hours per day children spent engaging in (1) recreational and (2) educational screen time.

#### Covariates

A range of confounding variables and competing exposures was derived based on existing literature, using the Daggity software^27^ (see directed acyclic graphs (DAGs) in online supplemental figure). Respondents completed questions about socio-demographic and geographical characteristics. From these, we derived child age, child sex, child ethnicity, child location (urban/rural/town and fringe), child learning disability, child physical disability and social grade. We derived the hours/week children spent in formal childcare based on parent report.^26^ A measure of parent mental distress was derived using the Kessler 6,^28^ summing the 6-item scale (responses on a 5-point Likert scale) to give a single score ranging from 0 to 24, with higher scores indicating higher respondent distress.^28^

### Data management

All data was provided in a fully anonymised form from YouGov UK. For data analysis, participants were identified by their unique anonymised identification number. The dataset were password-protected and held on a personal password-protected laptop.

### Patient and public involvement

Parents of preschool-aged children were involved in the development of the study questionnaire but did not contribute to this manuscript or to dissemination of this research.

### Analysis

Analyses were carried out using Stata/SE V.17. We derived descriptive characteristics for the sample, and for each of the exposure and outcome variables. Missing data were negligible due to YouGov collection methods, but some participants (n=148) were excluded due to how the CPS was scored (eg, where parents answered unsure/not known for adventure level). Visual inspection of histograms for each outcome variable was undertaken and assumptions of normality were met. We also assessed correlations between exposure variables to ensure collinearity was not present (correlation between educational and recreational screen time r=0.27; between adventurous play and recreational screen time: r=0.07; adventurous play and educational screen time r=0.09, between adventurous and outdoor play r=0.66). We ran linear regression models to explore how each of the four exposure variables (ie, adventurous and outdoor play, recreational and educational screen time) was associated with four outcome variables (ie, internalising and externalising, positive and negative affect scores). We fitted models using the svyset command in Stata, which allowed us to use survey weights. We used DAGs (DAGitty software^27^) for each exposure—outcome relationship, and models were minimally adjusted for appropriate confounders and competing exposures; results are provided for direct effects only. Finally, we included an interaction term between each play and screen time exposure variable to determine whether there was a differential impact of play by levels of screen time on children’s mental health outcomes. Model fit was assessed using the ‘testparm’ command (compatible with the svy command in Stata), applying an adjusted Wald test, with an interaction term retained when model fit was significantly better with the term included (ie, a value of p<0.05). As the adventurous play exposure variable was derived using an adventure level of 2 or more, we also conducted sensitivity analyses using an adventure level of 3 or more to determine how this influenced our exposure-outcome association. Post hoc analyses were conducted to explore the association between total screen time and mental health outcomes, including testing interactions, as WHO guidelines recommended no more than 1 hour per day total screen time^9^ but made no distinction by type. Finally, interactions between outdoor play and educational screen time were undertaken to determine whether it is adventurous play or outdoor engagement that modifies the effect of screen time on mental health outcomes.

### Findings

Participants were 1166 parents or carers of children aged 2–4 years (mean (SD) 3.1 (0.8) years) living in Britain (England, Scotland or Wales); 1018 provided valid data for all variables and were included in analyses. Descriptive characteristics of the sample are presented in table 1.

**Table 1:** Descriptive characteristics of included participants (n=1018)

Characteristic (n (%)) unless stated	N	Per cent
Respondent sex		
Male	334	33
Female	684	67
Respondent age (years)		
18–24	49	5
25–34	307	30
35–44	617	61
45+	45	4
Relationship to child		
Mother	660	65
Father	318	31
Stepparent	20	2
Other	20	2
Respondent ethnicity		
White	836	82
Minority	182	18
Social class		
Middle class (ABC1)	660	65
Working class (C2DE)	358	35
Parent mental health score (mean (SD))	7.6 (5.1)	
Employment status		
Working full time	520	51
Working part time	275	27
Student/retired	20	2
Not working	152	15
Unemployed	51	5
Child sex		
Male	537	53
Female	481	47
Child age (years)		
2	298	29
3	365	36
4	355	35
Childbirth order		
First born	549	54
Second born	329	32
Third or more	140	14
Time in childcare (mean (SD))	21 (12.7)	
Child learning disability		
Yes	62	6
No	886	87
Prefer not to say	26	3
Do not know	44	4
Child physical disability		
Yes	22	2
No	952	94
Prefer not to say	22	2
Do not know	22	2
Region		
England	880	86
London	123	12
North	241	24
Midlands	167	16
East	97	10
South	252	25
Wales	48	5
Scotland	90	9
Location		
Urban	806	79
Town or Fringe	115	11
Rural	97	10

Parents reported that 2–4 years old spent approximately 12 hours per week playing outdoors and 22 hours per week playing adventurously (table 2). Most children engaged in more than 1 hour of recreational screen time per day, but less than 1 hour per day of educational screen time (table 2). When combined, 159 (14%) children had <1 hour/day; 308 (28%) had >1–2 hours/day, 320 (29%) had >2–3 hours/day and 314 (29%) had >3 hours/day of screen time on average per day. Children’s mental health outcomes (mean (SD), range) were as follows: externalising score: 7.4 (3.5), 0–18; internalising score: 4.3 (3.5), 0–17; PANASPA: 19.4 (3.4), 5–25; PANASNA: 9.0 (3.3), 5–25.

**Table 2:** Summary of exposure variables

Time spent playing (hours/week) (mean (SD))		
Outdoor play		12.3 (7.9)
Adventurous play		22.6 (15.5)
Screen time (hours/day) (n (%))	Recreational	Educational
<1	271 (27)	628 (61)
1–2	471 (46)	226 (26)
>2	276 (27)	124 (12)

### Associations between adventurous play, screen time and mental health outcomes

Table 3 shows model results for all analyses (including unadjusted models and models adjusted for identified confounds (see above)). There was a positive association between hours/week play outdoors and adventurous play and positive affect, such that higher levels of play were associated with higher positive affect scores in young children. Higher levels of adventurous play were also associated with lower internalising scores. No other significant associations were found between play and outcomes.

**Table 3:** Associations between play, screen time and mental health outcomes in British preschool-aged children (n=1018)

	SDQ externalising		SDQ internalising		PANASPA		PANASNA	
	Unadjusted	Adjusted	Unadjusted	Adjusted	Unadjusted	Adjusted	Unadjusted	Adjusted
	Beta (95% CI)		Beta (95% CI)		Beta (95% CI)		Beta (95% CI)	
Ad play* (hours/week)	−0.02 (−0.03 to −0.00)	−0.01 (−0.03 to 0.00)	**−0.02** **(−0.04 to −0.01**)	**−0.02** **(−0.03 to −0.01**)	**0.04** **(0.03 to 0.05**)	**0.04** **(0.03 to 0.05**)	−0.00 (−0.02 to 0.01)	−0.00 (−0.01 to 0.01)
R^2^	0.0029	0.1798	0.0025	–	0.0235	0.1714	0.0002	–
Playing out* (hours/week) R^2^	−0.02 (−0.05 to 0.01) 0.0011	−0.02 (−0.05 to 0.00) 0.1807	−0.02 (−0.05 to 0.01) 0.0001	−0.02 (−0.05 to 0.00) 0.2636	**0.06** **(0.03 to 0.09**) 0.0146	**0.07** **(0.04 to 0.10**) 0.1721	0.01 (−0.02 to 0.03) 0.0019	0.00 (−0.02 to 0.03) 0.0019
Recreational ST† (ref: <1 hour/day)								
1–2 hours	0.42	0.49	0.37	0.42	−0.18	−0.26	**0.68**	**0.77**
	(−0.13 to 0.98)	(−0.02 to 1.01)	(−0.18 to 1.25)	(−0.06 to 0.90)	(−0.72 to 0.36)	(−0.76 to 0.25)	**(0.22 to 1.15**)	**(0.32 to 1.22**)
>2 hours	**0.72**	0.56	0.74	**0.68**	−0.42	−0.40	**0.95**	**0.84**
	**(0.15 to 1.29**)	(−0.01 to 1.14)	(0.12 to 1.36)	**(0.12 to 1.25**)	(−1.06 to 0.22)	(−1.02 to 0.21)	**(0.38 to 1.52**)	**(0.29 to 1.38**)
R^2^	0.0217	0.1828	0.0844	0.2848	0.0204	0.1547	0.0724	0.2204
Educational ST† (ref: <1 hour/day)								
1–2 hours	**0.53**	0.33	**1.23**	**1.21**	**−0.55**	−0.34	**0.81**	**1.28**
	**(0.03 to 1.04**)	(−0.18 to 0.83)	**(0.69 to 1.77**)	**(0.38 to 2.05**)	**(−1.05** to **−0.04**)	(−0.80 to 0.12)	**(0.29 to 1.35**)	**(0.46 to 2.09**)
>2 hours	**1.65**	**0.76**	**3.15**	**3.73**	**−1.40**	**−0.88**	**2.68**	**3.69**
	**(0.97 to 2.34**)	**(0.03 to 1.48**)	**(2.39 to 3.91**)	**(2.37 to 5.08**)	**(−2.18 to −0.62**)	**(−1.69 to −0.06**)	**(1.93 to 3.43**)	**(2.29 to 5.09**)
R^2^	0.0093	0.2687	0.0025	0.1539	0.0132	0.2045	0.0057	0.1825
Interaction analysis (educational ST) adjusted								Adjusted
Ad play				0.00				**0.02**
				(−0.02 to 0.02)				**(0.01 to 0.04**)
1–2 hours × ad play				−0.02 (−0.04 to 0.01)				**−0.03** **(−0.06 to −0.00**)
>2 hours × ad play				**−0.07** **(−0.11 to −0.03**)				**−0.08** **(−0.12 to −0.04**)
R^2^				0.2966				0.2369

* Analyses adjusted for: parental MH, child ethnic group, age, sex and time spent in childcare, caregiver employment status, household income and urban/rural.

† Parental MH, child ethnic group, age, sex, physical disability, learning disability, time spent in childcare, caregiver employment status, household income and urban/rural; bold indicates CI do not cross 0.

MH, mental health; PANASNA, Negative Affect PANAS score; PANASPA, Positive Affect PANAS score; SDQ, Strengths and Difficulties Questionnaire.

Educational screen time of 1–2 or >2 hours per day (vs <1 hour) was associated with poorer mental health outcomes in preschool-aged children. Most consistently, children with >2 hours/day educational screen time had higher externalising and internalising scores, lower positive affect and higher negative affect. Higher levels of recreational screen time were also associated with higher internalising and negative affect scores compared with those who had <1 hour/day screen time.

**Figure 2:**
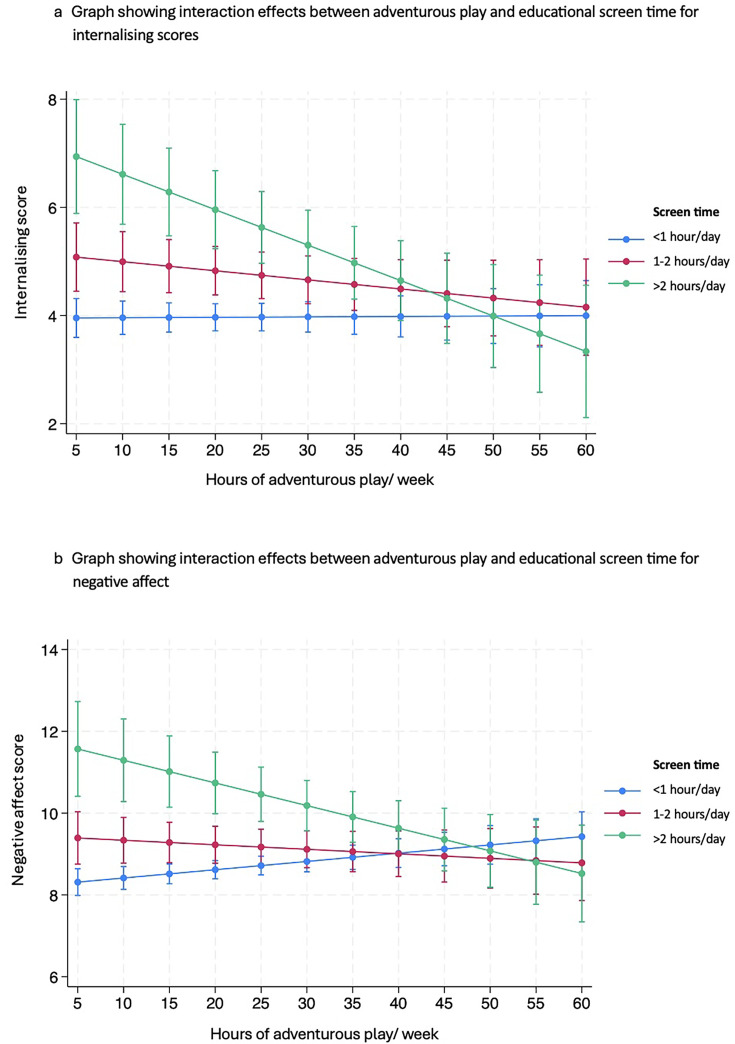
Graphs showing interaction effects between adventurous play and educational screen time for internalising and negative affect scores. Error bar indicates 95% confidence interval.

In addition, we identified significant interaction effects between adventurous play and educational screen time for internalising (*F*=6.16, p=0.0022) (figure 2a) and negative affect (*F*=8.47, p=0.0002) (figure 2b). For children in the higher educational screen time categories only, more time spent in adventurous play was associated with slightly lower overall internalising and negative affect scores, suggesting that impacts of screen time on mental health may be somewhat offset by higher levels of adventurous play.

We conducted sensitivity analyses to examine whether using an adventure level of >3 (compared with >2) affected associations with mental health outcomes: adventurous play remained associated with both internalising and positive affect (online supplemental table 2). In post hoc analyses, we explored the association between total screen time and mental health outcomes (online supplemental table 1). These showed that compared with children engaging in <1 hour/day of total screen time, higher levels of total screen time were associated with higher externalising scores (>3 hours/day: 0.79 (0.02–1.56)); internalising scores (>3 hours/day: 1.04 (0.37–1.71)); and negative affect (2–3 hours/day: 0.66 (0.08–1.23); >3 hours/day: 1.65 (0.97–2.33)), which align with the findings for educational screen time, with the exception of negative affect. No interactions were identified between total screen time and play variables. Finally, no interactions between outdoor play and educational screen time were identified, suggesting that general outdoor engagement, beyond adventurous play, does not modify the effect of screen time on mental health outcomes.

## Discussion

To our knowledge, this is the first study to assess the association between play, screen time and children’s mental health in preschool-aged children. As hypothesised, we found that higher levels of outdoor and adventurous play in 2–4 years old were associated with better mental health, and that screen time was associated with poorer mental health outcomes in these children. Adventurous play also appeared to moderate the association between educational screen time, internalising and negative affect. This work suggests that adventurous play could be one way to enhance children’s well-being and development in early childhood; cumulative high levels of screen time appear to be linked to negative mental health outcomes, even at this young age. Limiting young children’s screen time and encouraging adventurous play may be a viable means of improving children’s physical and mental well-being.

Evidence in school-aged children suggests that higher levels of adventurous play are associated with lower internalising scores and increased positive affect.^13^ We too found a positive association between adventurous play, internalising and positive affect here, such that more adventurous play was associated with better mental health outcomes. Though effect sizes were small, many factors potentially influence young children’s mental health,^7^ so this is not unexpected. Our results also held after adjusting for a range of plausible socio-demographic factors, indicating that findings are robust. In combination with prior work, it suggests that playing adventurously may offer positive benefits to children’s mental health from an early age.

Though not yet fully elucidated, several plausible mechanisms exist to explain this association. Exposing children to feelings of excitement and uncertainty early in life likely helps them to moderate their responses to external stimuli that are uncertain or provoke fear.^29 30^ Moreover, adventurous play often takes place outdoors and may allow for higher-intensity physical activity, both of which have been linked to better mental health in young children.^31^ While we identified an association between outdoor play and positive affect, we did not see the same association with internalising scores which was apparent in older-aged children,^13^ nor did we see interaction effects identified for adventurous play. More work is now required to understand how these differing types of play influence children’s mental health, including taking possible reverse causation into account.

Compared with 2–4 years old who are reported to engage in less than 1 hour of educational screen time per day on average, children with two or more hours of educational screen time had higher internalising, externalising and negative affect scores and lower positive affect scores. This is similar to findings from a large sample of USA 2–5 years old, where screen time of >1 hour/day was associated with decreased psychological well-being across a number of domains, including lower self-control, lower emotional stability and being more difficult to care for.^18^ Interestingly, the majority of associations were identified between educational screen time and mental health outcomes here. The distinction between screen time type was made to better understand the volumes of screen time young children were engaging in, given potential differing associated outcomes.^15^,^17^ It is important to consider that this distinction relies on parents’ interpretation of what applications and activities are educational, which is likely to differ between parents, given that many application-based games and programmes may be seen to have varying degrees of educational value.

Work conducted in a sample of UK parents of 0–18 months old suggested that limiting screen time was a low priority for parents,^32^ and that it is often used as a means by which parents can ‘get things done’.^33 34^ Screens are now ubiquitous in society, and no screen time for young children may be unrealistic, particularly with older siblings in the home. Nevertheless, our findings show that >1 hour/day spent on screens was negatively associated with young children’s mental health outcomes. This finding adds further weight to the WHO guidelines that recommend screen time be limited to <1 hour/day for under 5s,^9^ and aligns with research showing that screen time is linked to poorer physical health^19 35^ and sleep outcomes^20^ in young children. Levels of screen time appear to track across childhood, and associations between screen time and poorer mental health persist into later childhood and adolescence.^21^ Given these health implications, further work raising awareness of the potential harms of excess screen time in the early years may be required. Furthermore, it will be important for research to disentangle the impact that different types of screen time have so that evidence-based advice can be given to caregivers.

With many children spending more time on screens and evidence that outdoor, adventurous play is decreasing in some groups, we also sought to explore the potential interaction between adventurous play and screen time. In children with higher levels of educational screen time (>2 hours/day), those who also had higher levels of adventurous play had somewhat lower internalising and negative affect scores compared with their peers who also watched >2 hours/day of screen but had lower levels of adventurous play. This is interesting and indicates that adventurous play may in part mitigate some of the risks associated with high screen time to children’s mental health. This therefore adds to the growing evidence of the benefits of adventurous play, especially in the face of increasing exposure to screens.

It should be noted that as these data are cross-sectional, reverse causality cannot be ruled out. It is plausible that children with poorer mental health outcomes play less outdoors or adventurously, and/or that children with lower mood spend more time on screens. Moreover, parents of children who are more challenging or prone to hyperactivity may also facilitate screen time to provide downtime for their children. Nevertheless, given the similarities between our findings and those in older children, this work provides an important benchmark for further longitudinal and experimental work to explore how promoting adventurous play and limiting screen time may impact health and well-being during early childhood.

### Strengths and limitations

This study had several strengths. It is one of the first to assess adventurous play, screen time and mental health outcomes together in preschool-aged children, doing so in a nationally representative sample. Based on 2021 census data, the sample is comparable to Britain’s population proportions (ie, England 85%, Wales 5%, Scotland 8%; our sample: England 86%; Wales 5%; Scotland 9%).^36^ Participants were sampled from diverse geographical locations, socioeconomic strata and ethnic groups; population-level demographic survey weights were used to ensure the sample was representative of British children, reducing the risk of sampling bias. It used robust validated tools to assess types and levels of children’s play and mental health outcomes, adjusting for parent mental health outcomes as evidence suggests that parental mental health may impact estimates of child mental health. It did, however, rely on parental report for play and screen time exposure variables and mental health outcome data. While parent-report is frequently used as a means of collecting large-scale national data, it provides only one perception of the child’s behaviours and mental health. While parents were asked to focus on time spent engaging in these activities outside of childcare, it is possible that where parents spent less time with their child, they necessarily provided only an estimate of time spent playing or engaging in screen time. We are also unable to draw conclusions about how play during childcare influences children’s mental health outcomes from this work. While this cross-sectional data provides a helpful indication of associations between play, screen time and mental health, longitudinal evidence is now required to elucidate causal mechanisms to inform future promotion efforts.

## Conclusions

This study explored how play and screen time are associated with 2–4 years old children’s mental health outcomes. It suggests that in British preschool-aged children, both outdoor and adventurous play were associated with better mental health, whereas screen time was associated with poorer mental health outcomes. Adventurous play, in outdoor settings, appears to be important for preschoolers’ mental health and well-being and may potentially offset the negative associations between educational screen time and mental health. More work is required to determine the direction of causality between play, screen time and children’s mental health; how these associations change over time; and whether adventurous play may be protective in the face of increasing exposure to screen time in our youngest children.

## Supplementary material

10.1136/bmjopen-2025-105101 online supplemental figure 1

10.1136/bmjopen-2025-105101 online supplemental table 1

## Data Availability

Data are available in a public, open access repository. Data are available upon reasonable request.
