# Supplementary material for: Are play and screen time associated with British preschoolers’ mental health? Cross-sectional findings from the British Preschool Children’s Play Survey
Source: BMJ Open. 2026 Jan 29;16(1):e105101. doi: 10.1136/bmjopen-2025-105101 (PMC12863345; doi:10.1136/bmjopen-2025-105101)

Supplementary Figures

KEY:


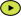
 exposure


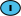
 outcome


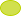
 ancestor of exposure


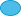
 ancestor of outcome


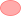
 ancestor of exposure *and* outcome


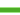
 causal path


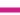
 biasing path

Supplementary Figure 1 – DAG used for analyses assessing the cross-sectional association between play variables and children’s mental health outcomes


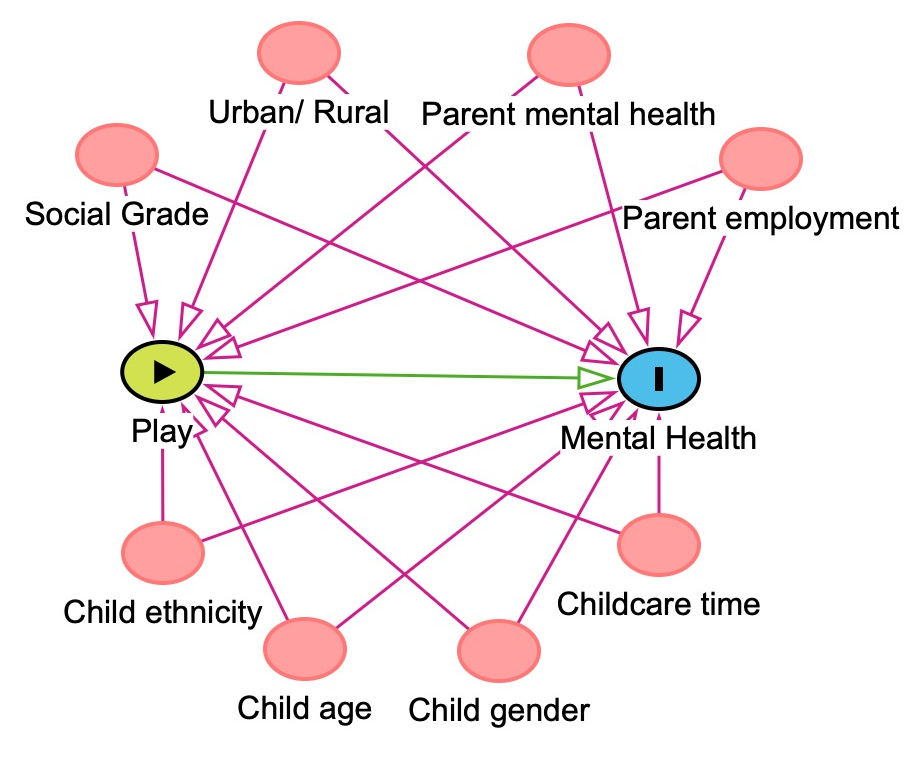


Supplementary Figure 2 – DAG used for analyses assessing the cross-sectional association between screentime variables and children’s mental health outcomes


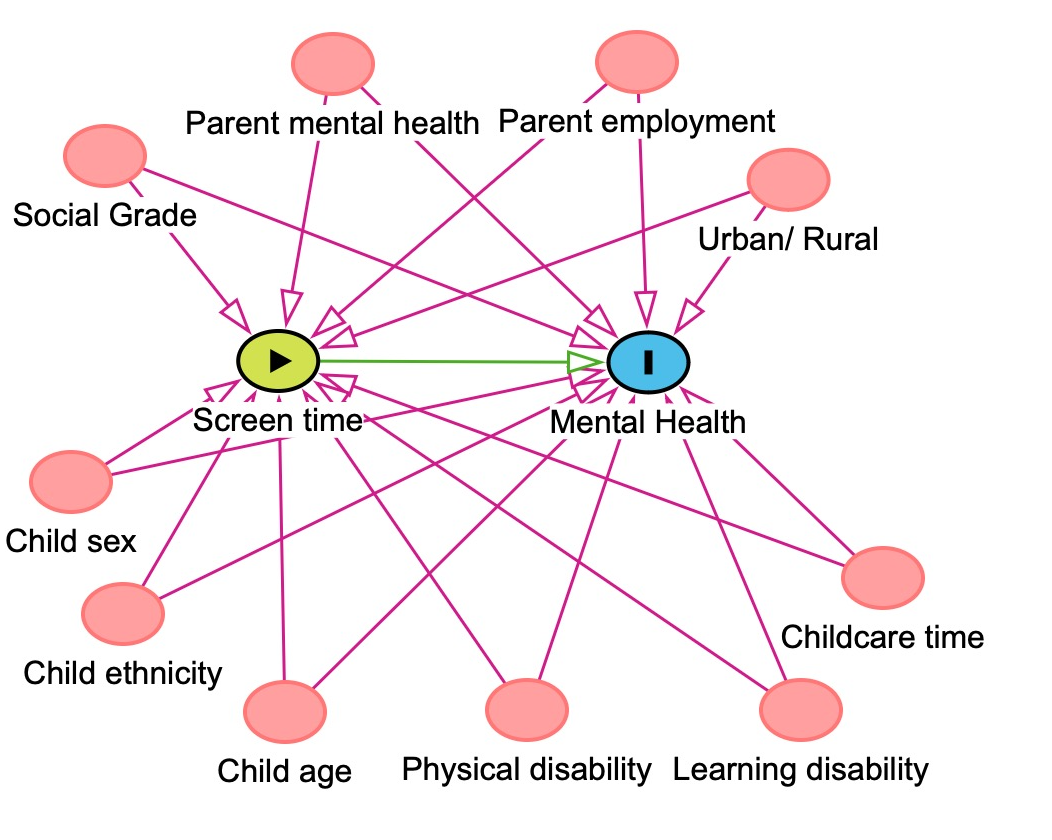

Supplement: online supplemental figure 1 [file bmjopen-16-1-s001.docx]
