# Supplementary material for: Are play and screen time associated with British preschoolers’ mental health? Cross-sectional findings from the British Preschool Children’s Play Survey
Source: BMJ Open. 2026 Jan 29;16(1):e105101. doi: 10.1136/bmjopen-2025-105101 (PMC12863345; doi:10.1136/bmjopen-2025-105101)
Supplement: online supplemental table 1 [file bmjopen-16-1-s002.docx]

| **Supplementary Table 1 – Associations between total screen time and mental health outcomes in children aged 2-4-years** | | | | | | | | | |
| --- | --- | --- | --- | --- | --- | --- | --- | --- | --- |
|  | SDQ Externalising | | SDQ Internalising | | PANAS PA | | PANAS NA | | |
|  | Unadjusted | Adjusted | Unadjusted | Adjusted | Unadjusted | Adjusted | Unadjusted | Adjusted | |
|  | Beta [95% CI] | | Beta [95% CI] | | Beta [95% CI] | | Beta [95% CI] | | |
| Ref <1hr/day |  |  |  |  |  |  |  |  | |
| 1-2 hrs/day | -0.13 | -0.31 | -0.34 | -0.38 | 0.03 | -0.11 | 0.30 | 0.33 | |
|  | [-0.81,0.55] | [-1.03,0.41] | [-0.97,0.29] | [-0.95,0.19] | [-0.70,0.77] | [-0.77,0.54] | [-0.21,0.80] | [-0.24,0.90] | |
|  |  |  |  |  |  |  |  |  | |
| >2-3 hrs/day | **0.85** | 0.69 | 0.59 | 0.49 | -0.19 | -0.27 | **0.72** | **0.66** | |
|  | **[0.16,1.55]** | [-0.06,1.45] | [-0.06,1.23] | [-0.10,1.08] | [-0.92,0.55] | [-0.94,0.39] | **[0.17,1.27]** | **[0.08,1.23]** | |
|  |  |  |  |  |  |  |  |  | |
| >3hrs/day | **1.44** | **0.79** | **1.85** | **1.04** | **-0.95** | -0.61 | **2.18** | **1.65** | |
|  | **[0.75,2.14]** | **[0.02,1.56]** | **[1.15,2.55]** | **[0.37,1.71]** | **[-1.72,-0.17]** | [-1.33,0.12] | **[1.54,2.82]** | **[0.97,2.33]** | |
| Analyses adjusted for parental MH, child ethnic group, age, physical disability, learning disability, time spent in childcare, screen time, care-giver employment status, household income and urban/rural; bold indicates CI do not cross 0. | | | | | | | | |  |

**Supplementary Table 2: Associations between adventurous play (level 3/+) and mental health outcomes**

|  | SDQ Externalising | | SDQ Internalising | | PANASPA | | PANASNA | |
| --- | --- | --- | --- | --- | --- | --- | --- | --- |
|  | Unadjusted | Adjusted | Unadjusted | Adjusted | Unadjusted | Adjusted | Unadjusted | Adjusted |
|  | Beta [95% CI] | | Beta [95% CI] | | Beta [95% CI] | | Beta [95% CI] | |
| Ad Play | -0.00 | 0.00 | **-0.02** | **-0.01** | **0.04** | **0.04** | 0.01 | 0.01 |
| (hr/week) | [-0.02,0.01] | [-0.01,0.02] | **[-0.03,-0.01]** | **[-0.03,-0.00]** | **[0.02,0.05]** | **[0.02,0.05]** | [-0.00,0.02] | [-0.00,0.02] |

Using adventure level >3: Mean=12.86 (SD 14.56) hours/week spent in adventurous play; analyses adjusted for parental MH, child ethnic group, age and time spent in childcare, care-giver employment status, household income and urban/rural.
