# Supplementary material for: Are play and screen time associated with British preschoolers’ mental health? Cross-sectional findings from the British Preschool Children’s Play Survey
Source: BMJ Open. 2026 Jan 29;16(1):e105101. doi: 10.1136/bmjopen-2025-105101 (PMC12863345; doi:10.1136/bmjopen-2025-105101)
Supplement: Supplementary file 3 [file bmjopen-2025-105101.reviewer_comments.pdf]

PEER REVIEW HISTORY

BMJ Open publishes all reviews undertaken for accepted manuscripts. Reviewers are asked to complete a checklist review form and are provided with free text boxes to elaborate on their assessment. These free text comments are reproduced below.

ARTICLE DETAILS

Title (Provisional)

Are play and screen time associated with preschoolers' mental health? Cross-sectional findings from the British Preschool Children's Play Survey

Authors

Hesketh, Kathryn R.; Dodd, Helen F.

VERSION 1 - REVIEW

**Reviewer**	**1**
**Name**	**Lu, Congchao**
**Affiliation**	**Tianjin Medical University**
**Date**	**19-Jun-2025**
**COI**	**None**

Thanks for this opportunity to review this interesting manuscript.

This manuscript presents a timely and valuable investigation into play behaviors and screen time in preschool children, addressing an important gap in the early childhood development literature. The finding that adventurous play may mitigate the negative effects of educational screen time is particularly noteworthy and offers practical insights for parents, educators, and policymakers. While the study makes a meaningful contribution to the field, there are several areas where the manuscript could be strengthened to enhance its methodological rigor and contextual framing. Below, I provide specific suggestions for improvement:

Q1. Clarify Sample Representativeness

The manuscript highlights the use of population-level weights to ensure representativeness, which is a commendable approach. However, the subnational sample sizes (England: 880, Wales: 48, Scotland: 90) raise concerns about proportional representation. Specifically, the underrepresentation of Wales and Scotland relative to England could potentially bias the findings.

Please provide evidence that the survey weights adequately compensate for these imbalances. A brief comparison of the weighted sample to UK census data for children aged 2–4 years would help reassure readers of the sample's representativeness.

Q2. Expand Interaction Analysis

The manuscript provides valuable insights into the interaction between adventurous play and educational screen time. However, the broader role of outdoor play in moderating screen time effects remains unexplored. Given that outdoor play is independently associated with positive affect (Table 3) and prior research suggests that outdoor exposure may buffer against the negative effects of screen time, it would be beneficial to extend this analysis.

Please conduct sensitivity analyses to test the interaction between outdoor play and educational screen time. This would clarify whether general outdoor engagement, beyond adventurous play, modifies the effects of screen time on mental health outcomes.

Q3. Reframe Novelty Claims

Page 18-19. “This study had several strengths. It is first study to assess adventurous play, screen time and mental health outcomes together in preschool-aged children, doing so in a nationally representative sample. Participants were sampled from diverse geographical locations, socioeconomic strata, and ethnic groups, with population-level survey weights used to ensure the sample was representative of British children, reducing the risk of sampling bias.”

Conclusions “This is the first study to explore how play and screen time are associated with 2-4-year-old children’s mental health outcomes.”

Please modify the language to reflect the study’s novel contribution more accurately. For example: This study provides novel evidence on the interplay of adventurous play and screen time ...

Minor Revisions

The following minor adjustments would enhance the manuscript’s clarity and readability:

Table 1: Add units (e.g., “years”) to the “Caregiver age” column to ensure clarity.

Table 2: Reformat the table to include clear headers and improve readability.

Table 3: Label the “Adjusted Models” to clarify the covariates included in each model, which would aid in interpretation.

**Reviewer**	**2**
**Name**	**Lakicevic, Nemanja**
**Affiliation**	**Lomonosov Moscow State University, Faculty of Psychology**
**Date**	**01-Jul-2025**
**COI**	**None**

Strength and limitation section should b included in the discussion section i.e., at the end of the paper. Unless is required by the journal to design the abstract this way, please remove it where it belongs. Furthermore, strength and limitations sections pertains to the design of your manuscript, not generalized statemets.

Other than this, kudos to the authors.

Good job!

**Reviewer**	**3**
**Name**	**Emond, Jennifer A**
**Affiliation**	**Dartmouth College, Biomedical Data Science**
**Date**	**27-Oct-2025**
**COI**	**None**

Please (briefly) comment on the survey design to understand the sampling design of the study.

More details on the validation of the Children's Play Scale is needed. Additionally, the scale appears to exclude play during childcare. Please report the how often children are in childcare; is it likely that more adventurous play in child care is an important variable to consider?

Same feedback goes for the adventurous play – is that validated? I worry that is a subjective measure that is biased by the parent's perspective.

For the screen time measure: was "educational" screen time defined for parents, or did parents self-define that term?

"We ran linear regression models to explore how each of the four exposure variables (i.e. adventurous and outdoor play, recreational and educational screentime was associated with four outcome variables (i.e. internalising and externalising, positive and negative affect scores …" please confirm that the distributions of the dependent variables appeared sampled from a normal distribution.

"Model fit was assessed using the 'testparm' command, and an interaction term retained when model fit was significantly better with the term included…" please report the specific goodness of fit measure(s) and criteria used for that process. Please report p-values for testing the interaction term in the model (as one term, not just for one level of the interaction term).

" We conducted sensitivity analyses for the adventurous play variable, using an adventure level of 3/+ to determine how this influenced association…" That line is unclear as written.

What is the correlation between educational and recreational screen time? Given that educational screen time was more common, and that the results are consistent with total screen time, I suggest that the main analysis should be for total screen time and the supplemental analysis should separate out screen type (the current Table 3). Why? Again, recreational and educational screen time are likely highly correlated and I have doubts about the validity of parental report of "educational" screen time.

Please also report on the correlations between each play variable and screen time; those may be highly correlated and the linear regression model might suffer from collinearity problems (consider including appropriate tests for collinearity).

Please report R-squared values for each model.

VERSION 1 - AUTHOR RESPONSE

Reviewer 1	
This manuscript presents a timely and valuable investigation into play behaviors and screen time in preschool children, addressing an important gap in the early childhood development literature. The finding that adventurous play may mitigate the negative effects of educational screen time is particularly noteworthy and offers practical insights for parents, educators, and policymakers. While the study makes a meaningful contribution to the field, there are several areas where the manuscript could be strengthened to enhance its methodological rigor and contextual framing. Below, I provide specific suggestions for improvement:	Thank you for your positive comments and your suggestions to improve the manuscript.
Q1. Clarify Sample Representativeness The manuscript highlights the use of population-level weights to ensure representativeness, which is a commendable approach. However, the subnational sample sizes (England: 880, Wales: 48, Scotland: 90) raise concerns about proportional representation. Specifically, the underrepresentation of Wales and Scotland relative to England could potentially bias the findings. Please provide evidence that the survey	Thank you for raising this point. Based on census data (2021), the sample is comparable to Britain's population proportions (Census: England 85%, Wales 5%, Scotland 8%; our sample: England 86%; Wales 5%; Scotland; 9%). We have now added this information to the strengths and limitation section.<br><br>Sample weights are used to pull the sample back to the population averages, but our proportions are broadly in line with the UK

weights adequately compensate for these imbalances. A brief comparison of the weighted sample to UK census data for children aged 2–4 years would help reassure readers of the sample's representativeness.	averages for 2021. As per your suggestion, we have included this in the discussion (Pg18):<br>*Based on 2021 census data, the sample is comparable to Britain's population proportions (i.e. England 85%, Wales 5%, Scotland 8%; our sample: England 86%; Wales 5%; Scotland; 9%)[38]*
Q2. Expand Interaction Analysis<br>The manuscript provides valuable insights into the interaction between adventurous play and educational screen time. However, the broader role of outdoor play in moderating screen time effects remains unexplored. Given that outdoor play is independently associated with positive affect (Table 3) and prior research suggests that outdoor exposure may buffer against the negative effects of screen time, it would be beneficial to extend this analysis.<br><br>Please conduct sensitivity analyses to test the interaction between outdoor play and educational screen time. This would clarify whether general outdoor engagement, beyond adventurous play, modifies the effects of screen time on mental health outcomes.	Thank you for this suggestion – we undertook sensitivity analyses to test the interaction between outdoor play and educational screen time, for which there were no significant interactions. We have included the following in the Methods (Pg10):<br>*Finally, interaction analyses between outdoor play and educational screen time were undertaken for all outcomes to determine whether it is adventurous play or outdoor engagement that modifies the effect of screen time on mental health outcomes*.<br>and Results (Pg14):<br>*Finally, no interactions between outdoor play and educational screen time were identified, suggesting that general outdoor engagement, beyond adventurous play, does not modify the effect of screen time on mental health outcomes.*<br>We have also made reference to this in the discussion, outlining the need for more evidence as to what exactly is driving the associations we see with adventurous play only (Pg 16):<br>*Whilst we identified an association between outdoor play and positive affect, we did not see the same association with internalising scores which was apparent in older-aged children[13] , nor did we see interaction effects identified for adventurous play. More work is now required to understand how these differing types of play influence children's mental health, including taking possible reverse causation into account.*
Reframe Novelty Claims<br>Page 18-19. "This study had several strengths. It is first study to assess adventurous play, screen time and mental health outcomes together in preschool-aged children, doing so in a nationally representative sample. Participants were	We have modified this language as suggested to:<br><br>Discussion (Pg16):<br>*To our knowledge, this is the first study to assess the association between play, screen time and*

sampled from diverse geographical locations, socioeconomic strata, and ethnic groups, with population-level survey weights used to ensure the sample was representative of British children, reducing the risk of sampling bias."<br>Conclusions "This is the first study to explore how play and screen time are associated with 2-4-year-old children's mental health outcomes."<br>Please modify the language to reflect the study's novel contribution more accurately. For example: This study provides novel evidence on the interplay of adventurous play and screen time …	*children's mental health in preschool-aged children.*<br><br>Conclusion (Pg19):<br>*This study explored how play and screen time are associated with 2-4-year-old children's mental health outcomes.*
Table 1: Add units (e.g., "years") to the "Caregiver age" column to ensure clarity.	We have added this (Pg12).
Table 2: Reformat the table to include clear headers and improve readability.	We have now reformatted this table (Pg13).
Table 3: Label the "Adjusted Models" to clarify the covariates included in each model, which would aid in interpretation.	We have included the variables used in the adjusted models in the table footer (Pg15).
Reviewer 2	
Strength and limitation section should b included in the discussion section i.e., at the end of the paper. Unless is required by the journal to design the abstract this way, please remove it where it belongs. Furthermore, strength and limitations sections pertains to the design of your manuscript, not generalized statemets.	It is a journal requirement to have the strengths and limitations bullet section here. We have now revised this to include only strengths and limitations of the methods (rather than the whole paper).
Reviewer 3	
Please (briefly) comment on the survey design to understand the sampling design of the study.	Thank you for this suggestion. We have now included a flow diagram for the study recruitment and included some text in the methods to provide a better overview of the sample (Pg6):<br>*YouGov recruit panel members from a diverse range of sources to ensure diversity, and use active sampling, inviting members of their research panel via email to complete online surveys. According to YouGov records, 14,250 parents had a child aged 2-4-years, and were eligible to participate (Figure 1). On starting the online survey, participants were initially asked to indicate whether they were parents of children aged 2 to 4*

	years, and after obtaining electronic consent, respondents were provided with a link to access the survey.
More details on the validation of the Children's Play Scale is needed. Additionally, the scale appears to exclude play during childcare. Please report the how often children are in childcare; is it likely that more adventurous play in child care is an important variable to consider?	We have now included information about the CPS reliability. Face validity work was undertaken with participants when developing the measure. We have now included the following in the methods (Pg8):<br>*The CPS was created with input from experts and parents, including iterative revisions with the parent group. Whilst the measure has not been formally validated against time use diaries or geographic information systems, it does have good face validity (as judged by parent piloting) and reliability[26].*<br><br>We included the average time children spent in childcare in Table 1 and adjusted for this continuous variable in the analyses, which we outline in the methods and Table 3. By adjusting for this variable, we focus our research questions and analysis on play outside of childcare. Play during childcare may also be important but is not our focus. It is also more difficult for parents to report what their child does whilst in childcare, when they are not with them. We have added that we cannot make conclusions about play in childcare to the limitations section (Pg18):<br>*We are also unable to draw conclusions about how play during childcare influences children's mental health outcomes from this work*<br><br>Overall, this research speaks to the importance of children's play experiences when they are not in childcare and relates to public health messaging for parents and caregivers related to children's play.
Same feedback goes for the adventurous play – is that validated? I worry that is a subjective measure that is biased by the parent's perspective.	Adventurous play is measured as part of the CPS. As with all self-report measures, we cannot be sure that this is children's actual levels of adventurous play but it does reflect parent's perceptions of their child's level of adventurous play. This was included as a limitation in the discussion (Pg18).
For the screen time measure: was "educational" screen time defined for	We included the following question in the survey:

parents, or did parents self-define that term?	Generally, how much time does your child spend looking at a screen (e.g. TV screen, tablet, phone etc.)<br>a) for recreational/ communication purposes and b) for educational purposes. Parents answered separately for week days and weekend days.<br><br>We have included this information in the manuscript for clarity (Pg8).
"We ran linear regression models to explore how each of the four exposure variables (i.e. adventurous and outdoor play, recreational and educational screentime was associated with four outcome variables (i.e. internalising and externalising, positive and negative affect scores ..." please confirm that the distributions of the dependent variables appeared sampled from a normal distribution.	Yes, the outcome variables were normally distributed. We have included a statement about this in the methods (Pg9): *Visual inspection of histograms for each outcome variable was undertaken and assumptions of normality were met.*
"Model fit was assessed using the 'testparm' command, and an interaction term retained when model fit was significantly better with the term included..." please report the specific goodness of fit measure(s) and criteria used for that process. Please report p-values for testing the interaction term in the model (as one term, not just for one level of the interaction term).	We have now added this to the methods section (Pg10):<br>*Model fit was assessed using the 'testparm' command (compatible with the svy command in STATA), applying an adjusted Wald test, with an interaction term retained when model fit was significantly better with the term included (i.e. a value of p<0.05).*<br><br>We have now included the following in the results (Pg13):<br>*In addition, we identified significant interaction effects between adventurous play and educational screen time for internalising (F=6.16, p=0.0022) and negative affect (F=8.47, p=0.0002).*<br><br>We have also included two figures to illustrate the interaction effects, which we believe are helpful additions to the manuscript.
" We conducted sensitivity analyses for the adventurous play variable, using an adventure level of 3/+ to determine how this influenced association..." That line is unclear as written.	We have now rephrased this to (Pg10):<br>*As the adventurous play exposure variable was derived using an adventure level of 2 or more, we also conducted sensitivity analyses using an adventure level of 3 or more to determine how this influenced our exposure-outcome association.*

What is the correlation between educational and recreational screen time? Given that educational screen time was more common, and that the results are consistent with total screen time, I suggest that the main analysis should be for total screen time and the supplemental analysis should separate out screen type (the current Table 3). Why? Again, recreational and educational screen time are likely highly correlated and I have doubts about the validity of parental report of “educational” screen time.	As part of our pre-analysis checks, we looked at the correlation between screen time variables (r=0.27). We believe this is low enough to warrant analysing as different exposure variables, as per our original research questions. We included total screen time as a sensitivity analysis, as we also wanted to explore whether total rather than categories of screen time were associated with children’s outcomes (given the current WHO screentime recommendations recommend an overall total amount). As these variables are not highly correlated, we believe it is interesting to retain the separate analyses and we have done so.<br><br>Reliability of parents reporting the different types of screen time is a separate and important issue, which we address in the manuscript.
Please also report on the correlations between each play variable and screen time; those may be highly correlated and the linear regression model might suffer from collinearity problems (consider including appropriate tests for collinearity).	As per above, we tested these prior to undertaking the analyses. There is a very low correlation between play and screen time variables, though the correlation between adventurous and outdoor play is higher (as would be expected). We have now included an explanation of this in the text of the manuscript (Pg9): We also assessed correlations between exposure variables to ensure collinearity was not present (correlation between educational and recreational screentime r=0.27; between adventurous play and recreational screentime: r=0.07; adventurous play and educational screentime r=0.09, between adventurous and outdoor play r=0.66).
Please report R-squared values for each model.	We have now included these in Table 3.
